# New insights and a computational model for understanding induced motion revealed through novel variants of the Flying Bluebottle Illusion

**DOI:** 10.1177/20416695251344457

**Published:** 2025-06-17

**Authors:** Ryan E.B. Mruczek, Gideon P. Caplovitz

**Affiliations:** 1 8717 College of the Holy Cross, USA; 2 University of Nevada, Reno, USA

**Keywords:** visual illusion, motion perception, relative motion, induced motion, motion contrast

## Abstract

The Flying Bluebottle Illusion is a compelling example of how the perceived trajectory of moving objects can be greatly influenced by other motion sources in the visual scene. In this article, we present a series of simplified variants of the Flying Bluebottle Illusion in which the true motion of a target is a circular orbit around a central point. However, when a similar but offset orbiting motion trajectory is added to a set of surrounding inducers, the perceived trajectory of the target is drastically altered in both extent and direction. In other words, the perceived orbiting motion of the target is “pulled” and distorted by the orbiting motion of the inducers. For simplicity's sake, we refer to the illusory effect revealed by these dueling orbits as the Dueling Orbit Illusion. These simplified variants lend themselves to empirical study with resultant effects that can be readily modeled. Here, we present a series of case examples for how the parameters of the stimuli may be varied to yield predictable effects, describe a straightforward computational model for quantifying the magnitude of the contextual influence, and discuss how the model may be leveraged to gain insight into the phenomenon of induced motion across a range of within and between observer domains.

## How to cite this article

Mruczek, R.E.B., & Caplovitz, G.P. (2025). New insights and a computational model for understanding induced motion revealed through novel variants of the Flying Bluebottle Illusion, *i–Perception*, *16*(0), 1–14. https://doi.org/10.1177/20416695251344457

## Introduction

Induced motion is a visual phenomenon in which the perceived motion of a target item is influenced by other sources of motion simultaneously present in the visual scene. First reported by the Gestalt psychologist Karl Duncker (1929/1938), the empirical study of induced motion has continued to the present day. The Flying Bluebottle Illusion (Anstis & Casco, 2006) is a striking example of induced motion in which the perceived motion of a target (a bluebottle fly) is greatly influenced by moving the background (a close-up scene of a rose). The perceived trajectory in such induced motion displays can be readily understood in terms of the vector decomposition and integration of distinct motion signals simultaneously present in the visual scene (Johansson, 1950, 1974, 1975; Loomis & Nakayama, 1973). Here, we present a series of simplified variants of the Flying Bluebottle Illusion in which the trajectory of a target is a circular orbit around a central point. When a similar but offset orbiting motion trajectory is added to a set of surrounding inducers, the perceived trajectory of the target is drastically altered in both extent and direction.

For simplicity, we refer to the illusory effect revealed by these dueling orbits as the “Dueling Orbit Illusion.” This simplified configuration shares many similarities to and was in fact inspired by both the Flying Bluebottle Illusion and a whimsical variant called “Once Upon a Time” created by Marco Bertamini and Andrew Lang that was featured in the 2018 Best Illusion of the Year Contest. First presented in the 2019 Best Illusion of the Year Contest (as the “Rotating Circles Illusion”), preliminary empirical results derived from several of the variants described below were presented at the 2021 Annual Meeting of the Vision Science Society (Mruczek & Caplovitz, 2021). Analogous to these and other examples of induced and relative motion, the Dueling Orbits Illusion reveals two distinct phenomenological effects: first, the perceived trajectory of a centrally located target circle is radically altered by the motion of surrounding circles; second, the perceived spatial extent over which the central target circle moves can also be modulated. While the Dueling Orbits Illusion likely manifests from the same basic motion mechanisms underlying induced and relative motion, as we will demonstrate in the sections that follow, the simplified configuration readily lends itself to the empirical study and computational modelling of contextual effects on perceived motion across a wide range of within and between observer domains.

Through a series of self-evident movie demonstrations, we first present several variants of the Dueling Orbits Illusion, showing multiple types of perceived motion trajectories that can be reliably induced. We then discuss how the perceived path of the target can be predicted and modeled by combining information about its motion with respect to the viewer and relative motion with respect to the inducers (Agaoglu et al., 2015; Wade & Swanston, 1987). Finally, we demonstrate some contextual factors that influence the strength of the illusion and discuss the implications of the illusion for furthering our general understanding of visual perception and the human visual system. Together, these demonstrations reproduce and extend findings previously derived in the study of the Flying Bluebottle Illusion and related induced motion phenomena, and provide a framework for extending them even further.

## Variations on Dueling Orbits

To fully appreciate the illusory effect, it is important to note that in all of the demo movies the target is moving in a circular orbit around a central point, as shown in Movie 1. In every case, the only change is the presence and trajectory of the surrounding objects.

**Figure 1.**
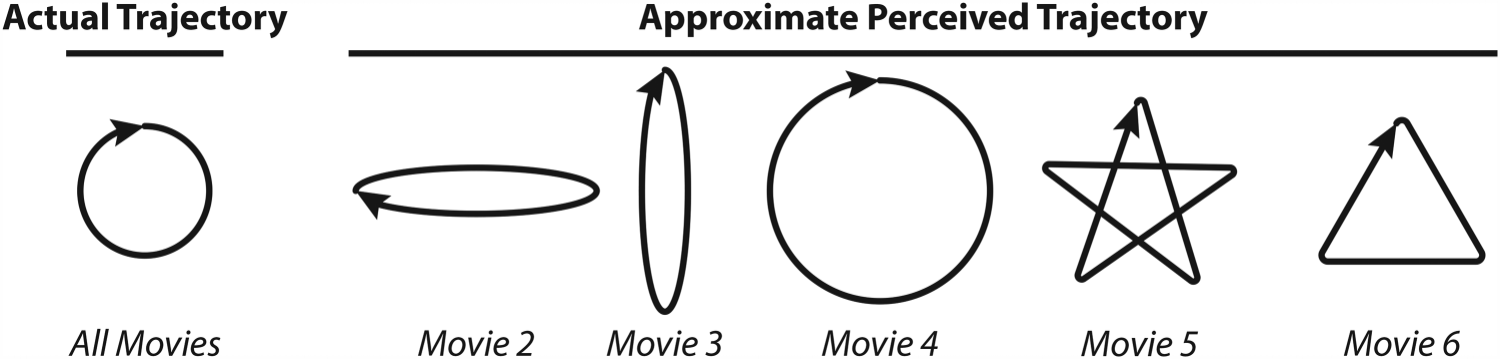
Comparison of the veridical (left, see Movie 1) and approximate perceived (right) motion trajectory of the central target for Movies 2–6. See Figure 2 for more details on predicting the perceived trajectory of the target under different viewing conditions.

**Movie 1.** In all movie demos in this article, the trajectory of the target is always a circular orbit around a central point (see Figure 1).

In the Dueling Orbits Illusion, the perceived motion of the central target is altered by the presence of four large inducers surrounding the target, each of which moves in its own orbital trajectory, with all four inducers moving synchronously. In Movie 2, the direction (counter-clockwise) of the inducers is opposite that of the target (clockwise). The speed (i.e., orbital period) of the target and inducers are matched, as is the phase (0°, both the target and inducers start from their highest orbital position. Although the actual trajectory of the target is circular, the perceived trajectory is strikingly elongated in the horizontal direction (Figure 1). In other words, the perceived vertical displacement of the target is greatly attenuated, whereas the perceived horizontal displacement is greatly enhanced. This is most apparent when the viewer tracks one of the inducers (magenta fixation spot on the upper inducer in Movie 2), but is also true when the viewer tracks the central target or maintains a stable peripheral fixation (green fixation spot in upper right of in Movie 2).

**Movie 2.** The central circle appears to move in a horizontally elongated ellipse (see Figure 1). The illusion is apparent when tracking the central target, tracking the upper inducer (magenta spot), or when fixating in a peripheral location (green spot).

The qualitative nature of the illusory trajectory can be altered in a highly predictable fashion by changing the relative phase, speed, or direction of the inducer motion relative to the target (Figure 1). This is illustrated in Movies 3–5, in which one or more motion components (phase, speed, or direction) are altered relative to the example in Movie 2.

In Movie 3, *the phase* difference between the inducers and target has been adjusted by 180° (i.e., the target starts in the highest orbital position, whereas the inducers start in the lowest orbital position). Doing so changes the perceived trajectory of the target to be largely vertical. In Movie 4, *the direction* of motion is the same for inducers and target (clockwise). This leads to a circular perceived trajectory of the target, but one that is expanded, appearing to have a greater eccentricity than its true motion. Finally, in Movie 5, *the speed* of the inducers is 1.5 times that of the target. This leads to a star-shaped perceived trajectory for the target. The target trajectory along the sides of the star appears quite straight, with relatively sharp changes in perceived trajectory at the corners. However, the precise shape of the perceived trajectory is somewhat dependent on eye position, which we discussed in more detail next.

**Movie 3.** The central circle appears to move in a vertically elongated ellipse (see Figure 1).

**Movie 4.** The central circle appears to move in an enlarged circle, or an orbit with an exaggerated eccentricity (see Figure 1).

**Movie 5.** The central circle appears to move in the shape of a 5-point star (see Figure 1).

## Eye Position

The above movies demonstrate a strong deviation in the perceived target trajectory from its veridical circular orbit in the Dueling Orbits Illusion. The illusory effect is also relatively robust to changes in eye position; an altered target trajectory is clear regardless of whether the viewer tracks the central target, tracks an inducer (magenta fixation spot), or maintains a stable peripheral fixation (green fixation spot). However, changes in eye position can lead to qualitative changes in the precisely perceived trajectory.

While the effect of eye position can be experienced by viewing Movies 2–5, it is perhaps best illustrated with the Dueling Orbits variant shown in Movie 6, in which the speed of the target is slowed to half that of the inducers. This yields a largely triangular perceived trajectory for the target (Figure 1). This is particularly striking when viewed with stable peripheral fixation (green fixation spot). In this case, the target trajectory along the sides of the triangle appears quite straight, with relatively sharp changes in perceived trajectory at the corners. Interestingly, when tracking the target itself, the triangular target trajectory appears to have rounded corners with slightly convex sides. In contrast, when tracking the upper inducer (magenta fixation spot), the triangular target trajectory appears to have slightly concave sides and a small loop at each corner. In the next section, we discuss how the perceived trajectory may be predicted from a weighted combination of multiple motion cues.

**Figure 2.**
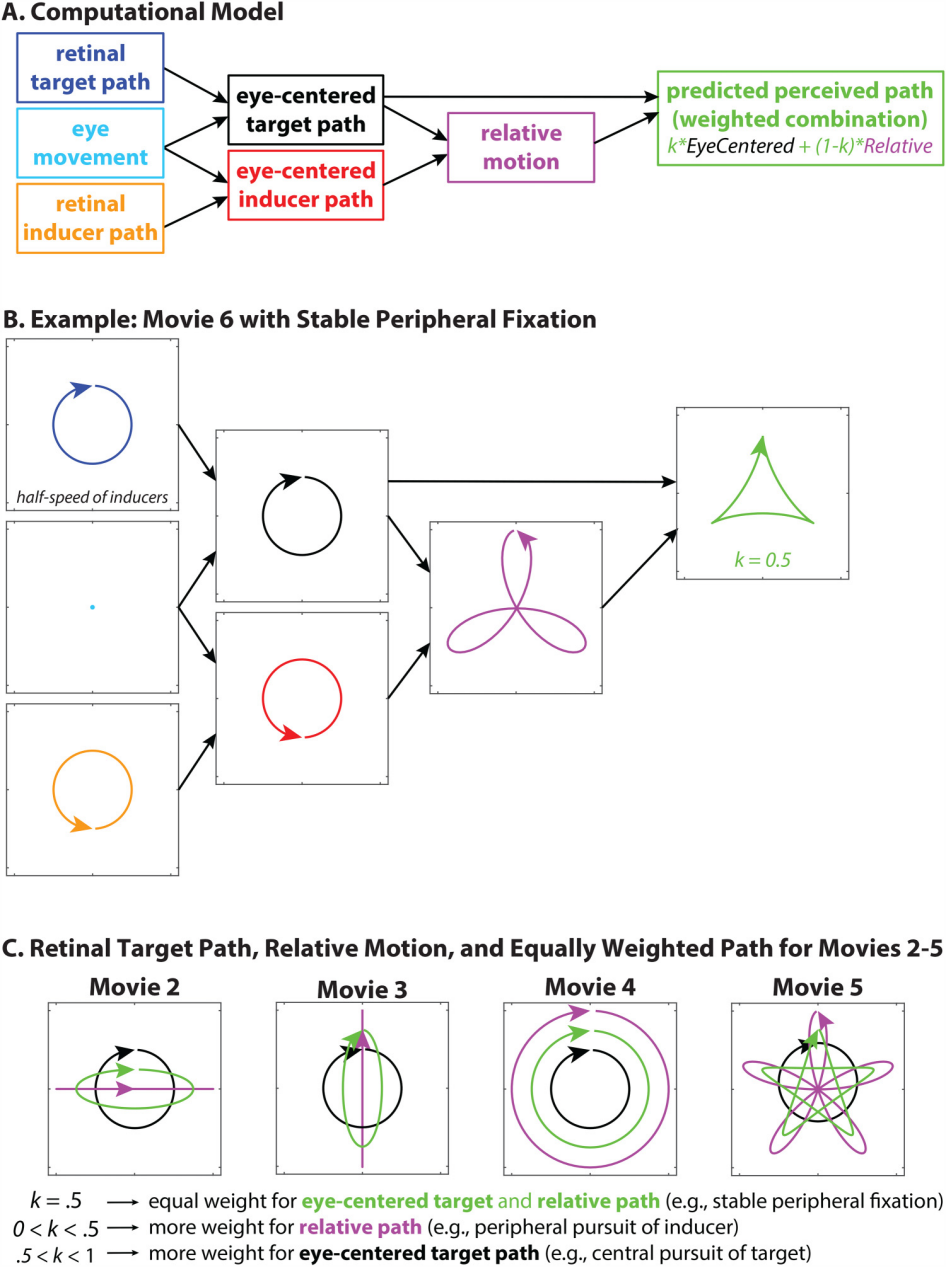
(a) A computational model showing how the perceived path of the target (green) is approximated by averaging the relative trajectory (magenta) and the eye-centered target path (black). (b) A detailed example for Movie 6, under conditions of stable peripheral fixation (green fixation spot in Movie 6). An equal weighting (*k* = .5) of the eye-centered and relative trajectories predicts the triangular perceived target path. (c) The eye-centered target path (black), relative path (magenta), and equally-weighted average (green, *k* = .5) for Movies 2–5. Pursuit of the target biases the perceived path in favor of the eye-centered path (.5 < *k* < 1), whereas pursuit of an inducer biases the perceived path in favor of the relative path (0 < *k* < .5). Note that the actual path of the speed, direction, and phase of the inducers and target differs across movies, but this is accounted for in the relative trajectory trace.

**Movie 6.** The central circle appears to move in a triangular trajectory, the exact shape of which depends on viewing position (see Figures 1 and 2).

## Predicting Perceived Trajectory

Duncker proposed a distinction between observer-relative and object-relative motion, both of which contribute to perceived motion (Duncker, 1929/1938; Wallach, 1959). This idea has been formally developed in quantitative models of motion perception (Agaoglu et al., 2015; Wade & Swanston, 1987), which propose a weighted combination of signals arising from different reference frames (e.g., retinal image motion, head and eye movements, relative motion). In the Dueling Orbits Illusion, the perceived trajectory of the target can be approximated by a weighted combination of its eye-centered path (i.e., its geocentric circular orbit assuming eye movements are the only self-motion) and its relative motion with respect to the inducers.

This model is illustrated in Figure 2(a). The eye-centered target path (black box) is calculated from a comparison of the trajectory of the target on the retinal (i.e., the retinal target path; blue box) and an efference copy of the movement of the eyes (cyan box). A similar calculation yields the eye-centered inducer path (red box) from a comparison of the retinal inducer path (orange box) and eye movements. Assuming perfect information (and no head or body movements), the perceived target trajectory would match the eye-centered target path (i.e., the eye-centered target, in principle, should match the trajectory of the target in an exocentric coordinate system). However, given uncertainty in the neural representation of the retinal target path and imperfections in fixation and/or smooth pursuit eye movements, the perceptual system may incorporate contextual cues about the relative motion of the target and inducers. The relative motion of the target with respect to the inducers (magenta box) is the difference between the eye-centered path of the target (black box) and inducers (red box).

The approximate perceived trajectory (green box) is predicted by taking a weighted average of the eye-centered target and relative motion trajectories (Figure 2(a)):

k×EyeCentered+(1−k)×Relative

Furthermore, how much the eye-centered and relative paths are weighted (i.e., the value of the proportion *k*) seems to depend on eye position. This is especially apparent for the parameters of Movies 5 and 6. As noted above (see Eye Position), viewing Movie 5 from a stable peripheral location leads to a largely triangular perceived trajectory, with straight edges and relatively sharp corners. This is close to what is predicted by an equal weighting of the relative path and real-world path (*k* = .5), as shown in Figure 2(b). In contrast, tracking the target causes the perceived trajectory to appear more rounded (but still with three clear bulges), as if the relative motion is being discounted in the calculation of perceived trajectory (.5 < *k* < 1). In a complementary fashion, tracking an inducer causes the perceived trajectory to appear biased toward the relative path (0 < *k* < .5), with loops at the corners like a three-petal flower. A similar pattern of results is apparent in Movies 2–4, and especially Movie 5 (Figure 2(c)).

To summarize, a weighted combination of multiple cues in different reference frames for motion perception helps explain why the Dueling Orbits Illusion persists, but with qualitative differences, across changes in eye position. A potential framework for understanding how and why the weighting changes in the way described above is provided by Bayesian cue integration (Angelaki et al., 2009; Kersten et al., 2004; Knill & Pouget, 2004). When tracking the target, eye movement signals can more reliably provide information about the eye-centered trajectory of the target. When tracking an inducer, the relative motion between target and inducer is given directly by the retinal trajectory of the target. In both cases, the more reliable signal has a stronger influence on the final perception. Under peripheral viewing, the two cues appear to be weighted roughly equally.

The degree to which weights are assigned to one factor or another could be empirically measured by examining the perceived shape of the target trajectory on a trial-by-trial basis in an individual observer. For example, the aspect ratio of the perceived elliptical trajectory in Movies 2 and 3 is a direct measure of the relative weights the observer is putting on the relative path and eye-centric trajectories of the target. This provides a powerful and remarkably accessible framework for examining what contextual factors can influence the process of cue integration and the basis of within and between observer variability in how cues are integrated.

## Additional Factors

The strength of the illusion is subject to additional contextual factors. In particular, the presence of a stable frame between the inducers and the target effectively eliminates the illusory effect (second half of Movie 7), even when the eyes are tracking an inducer. This is not true if the frame is placed beyond the inducers (first half of Movie 7), which has little effect on the strength and quality of the illusion.

**Movie 7.** The illusion is eliminated when a stable frame is placed between the target and inducers (second half of movie), but not when a stable frame surrounds the entire stimulus (first half of movie).

The illusion is also largely mitigated by stable central fixation (Movie 8). One possibility is that the central fixation point serves a similar purpose as a nearby central frame, providing a stable anchor point with which to compare the motion of the central target. We hypothesize a central fixation point or stable frame close to the target strengthens the positional certainty of the target (Saleki et al., 2021), leading to a higher degree of positional certainty regarding the eye-centered trajectory of the target and a discounting of the relative motion.

**Movie 8.** The illusion is abolished under central fixation (yellow spot).

The illusion is modulated by attentional selection (Gogel & Sharkey, 1989; Tse et al., 2013). Movie 9 includes two sets of inducers that are 180° out of phase with one another (i.e., the white inducers start at the bottom of their orbit, whereas the black inducers start at the top). While tracking the central gray target, one can attend either to the white or black inducers. This modulates, albeit weakly, the perceived trajectory of the target. When attending to the white inducers, the perceived trajectory of the target is elongated in the vertical direction. When attending to the black inducers, the perceived trajectory of the target is elongated in the horizontal direction. The more successful the observer is at covertly attending to one set of inducers or the other will determine how strongly the perceived trajectory is modulated.

**Movie 9.** Attention to one set of inducers, white or black, biases the perceived trajectory of the central light gray target.

Finally, we note that there is nothing special about using circles as the objects of the Dueling Orbits Illusion in the movies presented thus far. Indeed, Anstis and Casco (2006) demonstrated similar effects with their Flying Bluebottle Illusion using a fly as the target and multiple picture and textured backgrounds. As a tribute to their work, we illustrate this fact in Movie 10.

**Movie 10.** The Dueling Orbits Illusion can be generated with any objects. Here, a central fly appears to spiral around between inducers composed of a flyswatter and the face of Stuart Anstis, who, along with Clara Casco, first described the related Flying Bluebottle Illusion (Anstis & Casco, 2006).

## Discussion

Perceiving the motion path of an object requires a reference with which to compare its position over time. If the reference frame itself is moving, this can lead to the illusory changes in the speed, direction, or position of the target object, an effect termed induced motion (Duncker, 1929/1938; Wallach, 1959). This effect has also been referred to as motion contrast, relative motion, or relational motion (Mack, 1986; Reinhardt-Rutland, 1988; Wade & Swanston, 1987).

The Dueling Orbits Illusion is a striking demonstration of induced motion in which not only the direction but also the extent of the perceived target trajectory is altered. Most studies of induced motion have used a darkened room with minimal competing visual stimuli or reference frames (Reinhardt-Rutland, 1988), whereas the movie demonstrations presented here are of a small extent and can be viewed in any lighting. Indeed, incorporating an additional static cue beyond the inducers within the video frame itself does not disrupt the illusion (Movie 7). Overall, although others have reported a large intraobserver variability in susceptibility to the Duncker illusion (Mack, 1986; Zivotofsky, 2004), we expect that the illusory target trajectories illustrated in Figure 1 will be readily apparent to the vast majority of viewers in the presented movies.

Anstis and Casco (2006) described a similar illusion that they named the Flying Bluebottle Illusion, in which a target object moves in a circular orbit on a circularly translating background image. Indeed, the Dueling Orbits Illusion was inspired by and should be considered a simplified variant of their illusion, in which the background image is replaced by a small number of synchronously moving inducers. As such, the demonstrations presented above replicate and extend this work. Anstis and Casco (2006) observed that altering the relative phase of the target and background could lead to changes in the perceived size (see Movie 4) and shape (see Movie 5) of the target trajectory. They also reported that the circular trajectory could be perceived as a highly elongated, and nearly linear (7.5:1 or 14:1 ratio of ellipse length to height), trajectory (see Movies 2 and 3). Extending this work, our results show that a full-field texture background, like that used in the Flying Bluebottle Illusion, is not necessary and similar effects can be observed when the target is framed by a small number of synchronously moving inducers. We also demonstrate the role of eye-position and eye-movements (Movies 2–6 and 8), the mitigating effects of a stable frame (Movie 7), and that the illusion is subject to attentional modulation (Movie 9). The attentional-finding contributes to the hypothesis that induced motion is a relatively high-level visual phenomenon (Falconbridge et al., 2022). The movies presented here also illustrate various complex perceived trajectories that are possible under this framework (e.g., Movies 5, 6, and 10), thereby demonstrating the robust nature and high generalizability of the effect.

The robustness of this illusion may be due to a number of factors. First, the target itself is moving, which has been noted to enhance the effect relative to a stationary target (Reinhardt-Rutland, 1988; Wallach et al., 1978). Second, the target is well-framed by the inducers. Work by Cavanagh and colleagues on frame-induced position shifts has also shown the powerful effects of moving frames on the perceived position of a surrounded target (Cavanagh et al., 2022; Özkan et al., 2021; Whitney & Cavanagh, 2000). Third, the nature of the orbital motion of the target and inducers is circular and continuous. Objects with non-linear circular motion may be more susceptible to contextual influence given more uncertainty in their true motion (e.g., relative to purely vertical or horizontal motion), consistent with Bayesian models of motion perception (Kwon et al., 2015). Additionally, the continuous nature of the orbital motion may allow contextual influences on predictive motion extrapolation (Hogendoorn, 2020) to build up over time. Overall, these properties may account for the large illusory effects observed in the Dueling Orbits stimulus configurations.

Models of motion perception that offer an explanation of induced motion posit a weighted combination of signals arising from different reference frames (Agaoglu et al., 2015; Duncker, 1929/1938; Reinhardt-Rutland, 1988; Wade & Swanston, 1987; Wallach, 1959). Agaoglu et al. (2015) found that relative motion dominates for perception, although its influence falls off with increasing distance between the target object and the inducing object. Qualitatively, we find that the perceived target path is approximated by a weighted combination of relative motion and geocentric motion (accounting for retinotopic and spatiotopic frames in Agaoglu et al.'s terminology), and that the contribution of each is scaled by its reliability due to factors such as eye position. This leads to the testable prediction that specific weights depend on the precision (or lack of noise) in the neural representation of the target position and motion. For example, how strongly the relative motion influences the perceived target trajectory may depend on the viewer's ability to maintain stable fixation or on their pursuit gain. We expect that the illusory strength will largely follow the uncertainty in the neural representation of the target trajectory, given that the large and synchronized inducers should lead to a more robust neural representation of inducer motion.

The historical study of induced motion, like that of many perceptual phenomena, transitioned from the use of mechanical, analog apparatuses typical of the Gestalt-era to the digital, computerized formats ubiquitously in use today. The nuances of display format can influence how particular induced motion stimuli are perceived and inform our understanding of the mechanism underlying the phenomena (Léveillé et al., 2014; Léveillé & Yazdanbakhsh, 2010). As such, the Dueling Orbit Illusion and corresponding model presented here may have a specific application to the user experience in virtual- (VR) and particularly augmented-reality (AR) systems. Consider, for example, the case of a target presented in an augmented reality display against retinal motion arising from the real-world environment (e.g., while driving). The framework we have laid out here could be applied to identify if and how susceptible an individual may be to the effects of induced motion. Such motion-related individuation offers the potential to generalize to other domains of interest within the VR/AR community, such as motion sickness (Halow et al., 2023). Overall, given the subjectively strong effects demonstrated here, the Dueling Orbits Illusion may be useful in future studies of the factors that influence the weighting of different cues for motion perception.

## References

[bibr1-20416695251344457] Agaoglu M. N. Herzog M. H. Öğmen H. (2015). The effective reference frame in perceptual judgments of motion direction. Vision Research, 107, 101–112. 10.1016/j.visres.2014.12.009 25536467

[bibr2-20416695251344457] Angelaki D. E. Gu Y. DeAngelis G. C. (2009). Multisensory integration: Psychophysics, neurophysiology, and computation. Current Opinion in Neurobiology, 19(4), 452–458. 10.1016/j.conb.2009.06.008 19616425 PMC2749464

[bibr3-20416695251344457] Anstis S. Casco C. (2006). Induced movement: The flying bluebottle illusion. Journal of Vision, 6(10), 8. 10.1167/6.10.8 17132080

[bibr4-20416695251344457] Cavanagh P. Anstis S. Lisi M. Wexler M. Maechler M. R. ‘t Hart B. M. Shams-Ahmar M. Saleki S. (2022). Exploring the frame effect. Journal of Vision, 22(12), 5. 10.1167/jov.22.12.5 PMC9648424 36322075

[bibr5-20416695251344457] Duncker K. (1938). Induced motion (W. D. Ellis, trans.). In Ellis W. D. (Ed.), A source book of gestalt psychology (pp. 161–172). Routledge and Kegan Paul. (Original work published 1929).

[bibr6-20416695251344457] Falconbridge M. Hewitt K. Haille J. Badcock D. R. Edwards M. (2022). The induced motion effect is a high-level visual phenomenon: Psychophysical evidence. i-Perception, 13(5). 10.1177/20416695221118111 PMC9459461 36092511

[bibr7-20416695251344457] Gogel W. C. Sharkey T. J. (1989). Measuring attention using induced motion. Perception, 18(3), 303–320. 10.1068/p180303 2798015

[bibr8-20416695251344457] Halow S. J. Hamilton A. Folmer E. MacNeilage P. R. (2023). Impaired stationarity perception is associated with increased virtual reality sickness. Journal of Vision, 23(14), 7. 10.1167/jov.23.14.7 PMC10750839 38127329

[bibr9-20416695251344457] Hogendoorn H. (2020). Motion extrapolation in visual processing: Lessons from 25 years of flash-lag debate. The Journal of Neuroscience, 40(30), 5698–5705. 10.1523/JNEUROSCI.0275-20.2020 32699152 PMC7380963

[bibr10-20416695251344457] Johansson G. (1950). Configurations in event perception; an experimental study. Almquist and Wiksells.

[bibr11-20416695251344457] Johansson G. (1974). Vector analysis in visual perception of rolling motion. Psychologische Forschung, 36(4), 311–319. 10.1007/BF00424568 4844180

[bibr12-20416695251344457] Johansson G. (1975). Visual motion perception. Scientific American, 232(6), 76–89. 10.1038/scientificamerican0675-76 1145169

[bibr13-20416695251344457] Kersten D. Mamassian P. Yuille A. (2004). Object perception as Bayesian inference. Annual Review of Psychology, 55(1), 271–304. 10.1146/annurev.psych.55.090902.142005 14744217

[bibr14-20416695251344457] Knill D. C. Pouget A. (2004). The Bayesian brain: The role of uncertainty in neural coding and computation. Trends in Neurosciences, 27(12), 712–719. 10.1016/j.tins.2004.10.007 15541511

[bibr15-20416695251344457] Kwon O. S. Tadin D. Knill D. C. (2015). Unifying account of visual motion and position perception. Proceedings of the National Academy of Sciences of the United States of America, 112(26), 8142–8147. 10.1073/pnas.1500361112 26080410 PMC4491751

[bibr16-20416695251344457] Léveillé J. Myers E. Yazdanbakhsh A. (2014). Object-centered reference frames in depth as revealed by induced motion. Journal of Vision, 14(3), 15. 10.1167/14.3.15 24618108

[bibr17-20416695251344457] Léveillé J. Yazdanbakhsh A. (2010). Speed, more than depth, determines the strength of induced motion. Journal of Vision, 10(6), 10. 10.1167/10.6.10 20884559

[bibr18-20416695251344457] Loomis J. M. Nakayama K. (1973). A velocity analogue of brightness contrast. Perception, 2(4), 425–428. 10.1068/p020425 4803951

[bibr19-20416695251344457] Mack A. (1986). Perceptual aspects of motion in the frontal plane. In Boff K. R. Kaufman L. Thomas J. P. (Eds.), Handbook of perception and human performance (Vol. 1, pp. 17–11). New York: Wiley.

[bibr20-20416695251344457] Mruczek R. E. B. Caplovitz G. C. (2021, May 26). The Orbiting Circles Illusion: Induced changes in the length and direction of motion trajectory [Virtual poster]. V-VSS Annual Meeting 2021. 10.1167/jov.21.9.2330

[bibr21-20416695251344457] Özkan M. Anstis S. Hart B. M. T. Wexler M. Cavanagh P. (2021). Paradoxical stabilization of relative position in moving frames. Proceedings of the National Academy of Sciences, 118(25), 1–8. 10.1073/pnas.2102167118 PMC8237653 34131080

[bibr22-20416695251344457] Reinhardt-Rutland A. H. (1988). Induced movement in the visual modality: An overview. Psychological Bulletin, 103(1), 57–71. 10.1037/0033-2909.103.1.57 3279445

[bibr23-20416695251344457] Saleki S. Cavanagh P. Tse P. U. (2021). A position anchor sinks the double-drift illusion. Journal of Vision, 21(6), 3. 10.1167/jov.21.6.3 PMC8196411 34106221

[bibr24-20416695251344457] Tse P. U. Reavis E. A. Kohler P. J. Caplovitz G. P. Wheatley T. (2013). How attention can alter appearances. In Handbook of experimental phenomenology (pp. 291–315). John Wiley & Sons, Ltd. 10.1002/9781118329016.ch12

[bibr25-20416695251344457] Wade N. J. Swanston M. T. (1987). The representation of nonuniform motion: Induced movement. Perception, 16(5), 555–571. 10.1068/p160555 3330196

[bibr26-20416695251344457] Wallach H. (1959). The perception of motion. Scientific American, 201(1), 56–61. 10.2307/24940329 13668570

[bibr27-20416695251344457] Wallach H. Bacon J. Schulman P. (1978). Adaptation in motion perception: Alteration of induced motion. Perception & Psychophysics, 24(6), 509–514. 10.3758/BF03198776 750993

[bibr28-20416695251344457] Whitney D. Cavanagh P. (2000). Motion distorts visual space: Shifting the perceived position of remote stationary objects. Nature Neuroscience, 3(9), 954–959. 10.1038/78878 10966628

[bibr29-20416695251344457] Zivotofsky A. Z. (2004). The Duncker Illusion: Intersubject variability, brief exposure, and the role of eye movements in its generation. Investigative Ophthalmology & Visual Science, 45(8), 2867–2872. 10.1167/iovs.04-0031 15277515

